# The Quaternary Lidocaine Derivative QX-314 Produces Long-Lasting Intravenous Regional Anesthesia in Rats

**DOI:** 10.1371/journal.pone.0099704

**Published:** 2014-06-16

**Authors:** Yi Zhao, Cheng Zhou, Jin Liu, Peng Liang, Daqing Liao, Yanfang Chen, Xiangdong Chen

**Affiliations:** 1 Department of Anesthesiology and Laboratory of Anesthesia & Critical Care Medicine, Translational Neuroscience Center, West China Hospital, Sichuan University, Chengdu, Sichuan, P. R. China; 2 Department of Anesthesiology, Union Hospital of Tongji Medical College, Huazhong University of Science and Technology, Wuhan, Hubei, P. R. China; The Hebrew University Medical School, Israel

## Abstract

**Background:**

The lidocaine derivative, QX-314, produces long-lasting regional anesthesia in various animal models. We designed this study to examine whether QX-314 could produce long-lasting intravenous regional anesthesia (IVRA) in a rat model.

**Methods:**

IVRA was performed on tail of rats. EC_50_ (median effective concentration) of QX-314 in IVRA was determined by up-and-down method. IVRA on tail of rats was evaluated by tail-flick and tail-clamping tests. For comparison between QX-314 and lidocaine, 60 Sprague-Dawley rats were randomly divided into 6 groups (n = 10/group), respectively receiving 0.5 ml of 0.5% lidocaine, 0.25% QX-314, 0.5% QX-314, 1.0% QX-314, 2.0% QX-314 and normal saline. To explore the role of TRPV1 channel in IVRA of QX-314, 20 rats were randomly divided into 2 groups (n = 10/group), respectively receiving 0.5 ml of 1% QX-314 and 1% QX-314+75 µg/ml capsazepine. Toxicities of QX-314 on central nervous system and cardiac system were measured in rats according to Racine's convulsive scale and by electrocardiogram, respectively.

**Results:**

QX-314 could produce long-lasting IVRA in a concentration-dependent manner. EC_50_ of QX-314 in rat tail IVRA was 0.15±0.02%. At concentration of 0.5%, IVRA duration of QX-314 (2.5±0.7 hour) was significantly longer than that of 0.5% lidocaine (0.3±0.2 hour, *P*<0.001). TRPV1 channel antagonist (capsazepine) could significantly reduce the effect of QX-314. For evaluation of toxicities, QX-314 at doses of 5 or 10 mg/kg did not induce any serious complications. However, QX-314 at dose of 20 mg/kg (1% QX-314 0.5 ml for a rat weighing 250 g) induced death in 6/10 rats.

**Conclusions:**

QX-314 could produce long-lasting IVRA in a concentration-dependent manner. This long-lasting IVRA was mediated by activation of TRPV1 channels. Evaluation of toxic complications of QX-314 confirmed that low but relevant doses of QX-314 did not result in any measurable toxicity.

## Introduction

INTRAVENOUS regional anesthesia (IVRA) was firstly described by August Bier in 1908 for regional anesthesia on hand and forearm [1]. Because of its convenience, reliability and cost-effectiveness [1], IVRA remains a common regional anesthetic technique for anesthesiologists, especially appropriate for operations on limbs. However, clinical application of IVRA is mainly limited by toxicities of local anesthetics, tourniquet pain and short analgesic duration after releasing of tourniquet [2], [3], [4]. To reduce requirement of local anesthetics and to prolong analgesic duration of IVRA, many adjuvant drugs have been combined with local anesthetics in IVRA [5], [6], [7], [8]. However, none of these adjuvant drugs was satisfied. Some of these adjuvant drugs were unable to significantly reduce the requirement of local anesthetics and some of them induced unacceptable complications [9], [10]. Therefore, it is potentially useful to find a local anesthetic that could produce long-lasting IVRA with acceptable side effects.

QX-314, a quaternary derivative of lidocaine, has been demonstrated to produce long-lasting regional anesthesia in various animal models because of its permanent positive charge [11]. Unfortunately, anesthetic potency of QX-314 is also attenuated by this positive charge because QX-314 is difficult to penetrate neural membranes [12]. Recently, activation of TRPV1 channels has been reported to specifically deliver QX-314 into nociceptor, so as to produce a rapid onset and long-lasting nociceptive-selective blockade [12], [13], [14]. Various TRPV1 channel agonists such as capsaicin and acid solution have been used to deliver QX-314 [12], [13], [14], [15], [16]. Interestingly, QX-314 alone at relatively high concentrations could activate TRPV1 channels [17]. Therefore, QX-314 at relatively high concentrations might produce a rapid onset and long-lasting IVRA without any adjuvant drug. It is still elusive whether QX-314 could produce effective IVRA.

Because quaternary derivative is difficult to penetrate blood-brain barrier [18], it is believed that QX-314 is ineffective to induce toxicities on central nervous system (CNS). In addition, for its high water solubility, cardiac toxicity of QX-314 might also be weak because toxicity of local anesthetics on heart positively correlates with their hydrophobicity [19]. However, a recent study indicated that CNS and cardiac toxicities of QX-314 were similar or even more serious than that of lidocaine [20].

In the present study, we hypothesized that QX-314 alone could produce long-lasting IVRA through activation of TRPV1 channels. All the experiments were designed to investigate this hypothesis. In addition, toxicities of QX-314 on CNS and cardiac system were evaluated at relevant doses.

## Materials and Methods

SD (Sprague-Dawley) rats weighing 200 to 300 g (evenly composed of males and females) were provided by Experimental Animal Center of Sichuan province. All the animals were housed in cages (two or three rats per cage) with free access to food and water, and were maintained on a 12/12 h light-dark cycle. All the experiments were performed from 08:00 to 14:00. All experimental protocols were approved by Institutional Animal Experimental Ethics Committee of Sichuan University (Chengdu, Sichuan, P.R. China), and the experiments were carried out in accordance with the Declaration of National Institutes of Health Guide for Care and Use of Laboratory Animals (Publication No. 80-23, revised 1996).

### Drugs and chemicals

QX-314 and capsazepine (TRPV1 channel antagonist) were purchased from Sigma-Aldrich Co. Ltd. (Shanghai, China). Lidocaine was purchased from Shanghai Fortune Zhaohui (Shanghai Fortune Zhaohui Pharmaceutical Ltd., Shanghai, China). Stock solution of capsazepine was prepared at concentration of 10 mg/ml, dissolved in dimethyl sulfoxide (DMSO). The final concentration of DMSO was lower than 1% for *in vivo* injection. QX-314 and lidocaine at various concentrations (w/v) were prepared by dilution with normal saline.

### Intravenous regional anesthesia (IVRA) model in tail of rats

**Figure 1:**
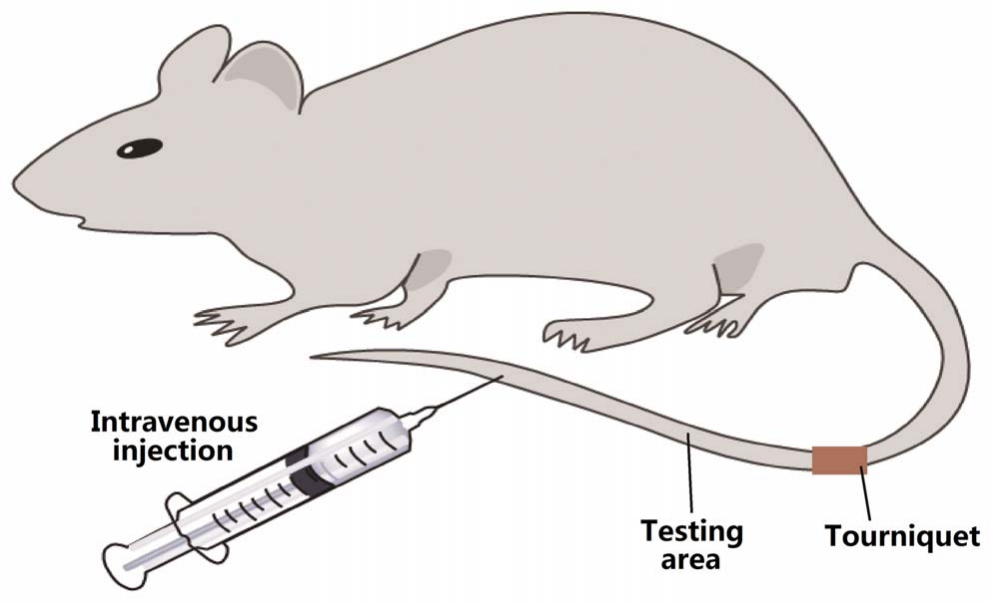
Venipuncture was performed on the distal third of the tail with a 24-gauge IV cannula (Terumo Medical Corp, Tokyo, Japan). The tail was then exsanguinated by a rubber strip. The exsanguination strip was released after an elastic rubber tourniquet. After the application of tourniquet, study drugs were intravenously injected at volume of 0.5-flick and Tail-clamping test were applied on testing area of the tail.

A rat model of IVRA developed by our laboratory [21] was used in the present study (Figure 1). Briefly, venipuncture was performed on the distal third of the tail with a 24-gauge IV cannula (Terumo Medical Corp, Tokyo, Japan). The tail was then exsanguinated by a rubber strip. The exsanguination strip was released after application of an elastic rubber tourniquet. After application of the tourniquet, study drugs were injected at volume of 0.5 ml for each rat, and the tourniquet was released 10 min later. After injection, rat tail IVRA was assessed by tail-clamping test and analgesic effect was evaluated by tail-flick test, as previously described [22]. All the evaluations were performed by a trained observer blinded to the group allocation and treatments of the rats.

In tail-flick test, tail-flick latency (the time from onset of radiant heat to tail-flick response) was measured by a tail-flick analgesia meter (Tail-Flick Unit 7360; Ugo Basile, Comerio, Italy). The intensity of radiant heat (parameter of the analgesia meter) was set at 90, and cut-off time of radiant heat was set at 10 seconds. The middle third of the tail distal to the tourniquet was the testing area. Baseline tail-flick latency was measured before venipuncture. The analgesic effect was standardized by calculating the percentage of maximum possible effect (%MPE) according to the following formula [22]:
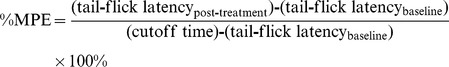



The time from end of injection to %MPE reaching 50% was defined as onset time of analgesia; the time from tourniquet releasing to %MPE less than 50% was defined as analgesic duration.

Tail-clamping test was performed one minute after each tail-flick test, using an alligator clip (10 A, type 85, length 2–1/8 in. with jaws that open maximally to 5/16 in.; Newark Electronics, Dublin, CA). Cut-off time of tail-clamping stimulus was 10 seconds. In naive rats, aversive responses (e.g. jerk, flinch and squeak) would be observed during application of tail-clamping stimulus. No aversive response to tail-clamping stimulus was defined as successful IVRA in rat tails. The time from end of injection to successful IVRA was defined as onset time of IVRA; the time from tourniquet releasing to re-appearance of aversive responses to tail-clamping stimulus was defined as duration of IVRA.

To compare IV regional anesthetic effect between QX-314 and lidocaine, 60 rats were randomly divided into 6 groups (n = 10/group), respectively receiving 0.5 ml of 0.5% lidocaine, 0.25% QX-314, 0.5% QX-314, 1.0% QX-314, 2.0% QX-314 and normal saline. After intravenous injection, percentage of successful IVRA (determined by tail-clamping test), onset time and duration of IVRA, onset time and duration of analgesia (determined by tail-flick test) were determined. To explore the role of TRPV1 channel in IVRA of QX-314, 20 rats were randomly divided into 2 groups (n = 10/group), respectively receiving 0.5 ml of 1% QX-314 and 1% QX-314+75 µg/ml capsazepine. After intravenous injection, percentage of successful IVRA, durations of IVRA and analgesia were determined.

After complete recovery from tail IVRA, all the rat tails were observed for two days to ensure whether injuries (e.g. change of skin color, ulcer, necrosis and infection) were induced.

### Determination of EC_50_ of QX-314 in rat tail IVRA

In the present study, EC_50_ (median effective concentration) of QX-314 in rat tail IVRA was measured using up-and-down method [22]. Successful IVRA (the positive endpoint in up-and-down method) was defined as no aversive response to tail-clamping stimulus. Based on our preliminary experiment, the concentrations of QX-314 were designed at 0.31%, 0.25%, 0.20%, 0.16%, 0.13% and 0.10%, respectively in up-and-down procedure. The initial concentration of QX-314 was set at 0.25% for the first rat. After application of tail-clamping stimulus, if no aversive response was observed, the concentration of QX-314 was decreased to a lower concentration for the next rat; if aversive responses were found, the concentration of QX-314 was increased to a higher concentration for the next rat. This up-and-down procedure was repeated until six crossovers (positive to negative or negative to positive effect) were obtained.

### Evaluation of toxicities of QX-314 on CNS and cardiac system

Toxicities of QX-314 on central nervous system (CNS) and cardiac system were determined by Racine's convulsive scale [23], [24] and ECG (Electrocardiography) recording [20], respectively. Venipuncture was performed on the tail of rats with a 24-gauge IV cannula (Terumo Medical Corp, Tokyo, Japan). QX-314 at doses of 5, 10 and 20 mg/kg (n = 10 for each dose) were intravenously injected *via* tail vein in a single bolus. Of note, in rat tail IVRA, if 0.5% QX-314 0.5 ml was injected into blood vessel without a tourniquet, the dose of QX-314 was 10 mg/kg for a rat weighing 250 g.

After injection of QX-314, toxicities of QX-314 on central nervous system was evaluated by convulsive behavior of the rats according to the scale modified from Racine's scale [23], [24]: 0 =  no convulsive behavior; 1 =  sedation, less activity but revival of righting reflex; 2 =  facial clonus or head nodding; 3 =  forelimb clonus; 4 =  rearing, animal in a standing posture aided by tail and laterally spread hand limbs showing in increased tone; 5 =  tonic-clonic seizure.

For evaluation of toxicity of QX-314 on cardiac system, rats were anesthetized by 2% isoflurane. Then the rats were placed on a board to keep four limbs stretched. Body temperature of rats was kept at 37–38°C. The three lead electrocardiographic (ECG) was applied and electrodes were placed at left upper limb, right upper limb and left lower limb, respectively. ECG data was acquired by a BL-410 biological function experimental system (Taimeng Science and Technology Ltd., Chengdu, Sichuan, China) equipped with AC (alternating current) and 100 Hz filters. The system was connected to the computer running the record software (BL-410). QX-314 at doses of 5, 10, 20 mg/kg (n = 10 for each dose) was intravenously injected *via* tail vein in a single bolus. ECG of the rats was recorded before (baseline) and three minutes after injection of QX-314. Toxicity of QX-314 on cardiac system was determined by change of heart rate after the injection of QX-314.

## Statistical Analysis

SPSS 16.0 (SPSS Inc., Chicago, IL) was used for all the statistical analysis in the present study except for particular mention. The sample size was 10 rats/group and all the rats were randomly allocated into each group. Values of results were expressed as mean ± SD. EC_50_ of QX-314 was calculated by averaging the concentrations of the six crossovers. For each time point in tail-flick experiment, analysis of variance with the Scheffe's test was applied for comparison among groups and least significant difference was used for the *post hoc* test. Repeated measures analysis of variance was applied to the entire duration. For tail-clamping data, (survival analysis) Kaplan-Meier method with log-rank test was applied among the groups. Kruskal-Wallis test followed by *post hoc* of Dunn's test (software Prism 6.0, Graph Pad, San Diego, CA) was applied for comparison of onset time and durations among the groups. Heart rate of the rats was expressed as mean ± SD and compared by one-way ANOVA followed by *post hoc* of Turkey's test. In all cases, *P*<0.05 was considered as statistically significant.

## Results

### QX-314 alone produced long-lasting IV regional anesthesia in tail of rats

**Figure 2:**
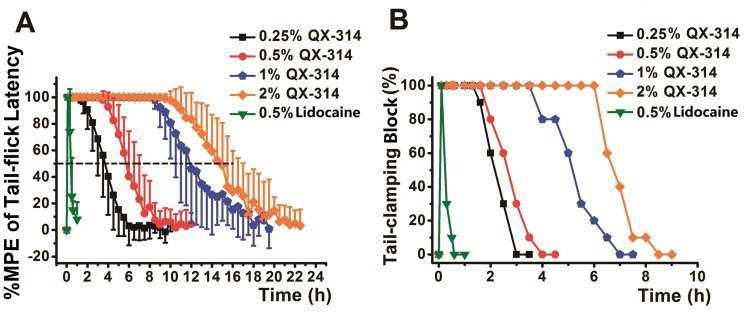
Intravenous regional anesthetic (IVRA) effect of QX-314 and lidocaine was evaluated by tail-flick and tail-clamping tests. A: analgesic effect of QX-314 and lidocaine was evaluated by tail-flick test. Tail-flick latency (%MEP) of all rats in the 5 groups (n  = 10/group) increased to 100%. The duration of analgesia was significantly longer in 0.5% QX-314 group than that of 0.5% lidocaine group (*P*<0.001). B: IVRA of QX-314 and lidocaine was evaluated by tail-clamping test. Normal saline did not produce any anesthetic relevant effect (Normal saline group was not shown in this figure).

QX-314 could produce long-lasting IVRA in a concentration-dependent manner. When successful IVRA was defined as no aversive responses of rats to tail-clamping stimulus, EC_50_ of QX-314 in rat tail IVRA was 0.15±0.02% (n = 20). With increasing of concentrations of QX-314, onset time of IVRA was significantly shortened and duration of IVRA was significantly prolonged. As shown in Figure 2 and Table 1, QX-314 at all the concentrations (0.25%-2.0%) produced analgesic effect and successful IVRA in tail of rats. For analgesic effect which evaluated by tail-flick test (Figure 2A), %MPE of tail-flick latency increased to 100% in all the rats from QX-314 and lidocaine groups. For IVRA which evaluated by tail-clamping test (Figure 2B), successful IVRA was induced in all the rats from QX-314 and lidocaine groups. At the same concentration of 0.5%, onset time of IVRA in 0.5% QX-314 group (10.4±2.7 min) was significantly delayed than that of 0.5% lidocaine group (1.0±0.0 min, *P*<0.001). However, duration of IVRA in 0.5% QX-314 group (2.5±0.7 hour) was significantly longer than that of 0.5% lidocaine group (0.3±0.2 hour, *P*<0.001). All the results were listed in Table 1.

**Table 1:** IVRA of QX-314 and lidocaine.

	0.25% QX-314	0.5% QX-314	1.0% QX-314	2.0% QX314	0.5% Lido
Analgesic onset (min)	10.5±3.1*	7.8±3.1*	6.2±2.9*	6.8±3.0*	1.0±0.0
Analgesic duration (hour)	3.9±1.0*	6.1±1.2*	12.6±2.8*	14.9±2.7*	0.5±0.2
Onset of IVRA (min)	13.5±3.3*	10.4±2.7*	9.5±3.5*	9.4±2.9*	1.0±0.0
Duration of IVRA (hour)	2.0±0.4*	2.5±0.7*	4.9±1.0*	6.8±0.9*	0.3±0.2

Results were expressed as mean ± SD. “Lido”: lidocaine. Analgesic effect was determined by tail-flick test and IVRA was determined by tail-clamping test.

*: compared to 0.5% lidocaine group, *P*<0.01

No obvious complications or toxicities were found after releasing of tourniquet in all the study rats. The rat tail IVRA produced by lidocaine and QX-314 was completely reversible and sensory function of all the study rats recovered. In addition, none of rats was found to develop injuries on tail skin (e.g. change of skin color, ulcer, necrosis and infection) during the two-day observation after recovery from tail IVRA.

### TRPV1 channel antagonist reduced IV regional anesthetic effect of QX-314

QX-314 at concentration of 1% could produce successful IVRA in all the rats (n = 10/group, Table 2). With co-injection of 1% QX-314+75 µg/ml capsazepine, successful IVRA was only induced in 2 out of 10 rats (Table 2). Meanwhile, duration of IVRA produced by 1% QX-314 was significantly reduced from 4.5±0.8 to 1.3±0.2 hour (*P*<0.001, Table 2) by adding 75 µg/ml capsazepine. In tail-flick test, analgesic duration of 1% QX-314 was also shortened from 10.9±2.5 to 3.5±1.0 hour (*P*<0.001, Table 2). All the results were listed in Table 2.

**Table 2:** Capsazepine attenuated IVRA of QX-314.

	Successful IVRA rate	Analgesic duration (hour)	Duration of IVRA (hour)
1% QX-314	100%	10.9±2.5	4.5±0.8
1% QX-314+CPZ	20%	3.5±1.0*	1.3±0.2*

Results were expressed as mean ± SD. “CPZ”: capsazepine.

*: compared to QX-314 alone, *P* <0.001

### Toxicities of QX-314 on CNS and cardiac system

No convulsive behavior (0 score in Racine's scale) was observed after injection of QX-314 at dose of 5 mg/kg in all the rats. After injection of QX-314 at dose of 10 mg/kg, no convulsive behavior was found in 2 out of 10 rats and 6/10 rats were scored 1 (sedation, less activity but revival of righting reflex) according to the Racine's scale, and 2 out of 10 rats were scored 2 (facial clonus or head nodding). After injection of QX-314 at dose of 20 mg/kg, 6 out of 10 rats died and 4 out of 10 rats were scored 5 (tonic-clonic seizure). All the results were listed in Table 3.

**Table 3:** Toxicity of QX-314 on central nervous system according to Racine's convulsive scale.

Doses (mg/kg)	0:Normal	1	2	3	4	5	Death	Total
5	10	0	0	0	0	0	0	10
10	2	6	2	0	0	0	0	10
20	0	0	0	0	0	4	6	10

For each dose of QX-314, 10 rats were observed. Behavior of rats was scored according to Racine's score scale including score 0-5, as previously described [23], [24].

**Figure 3:**
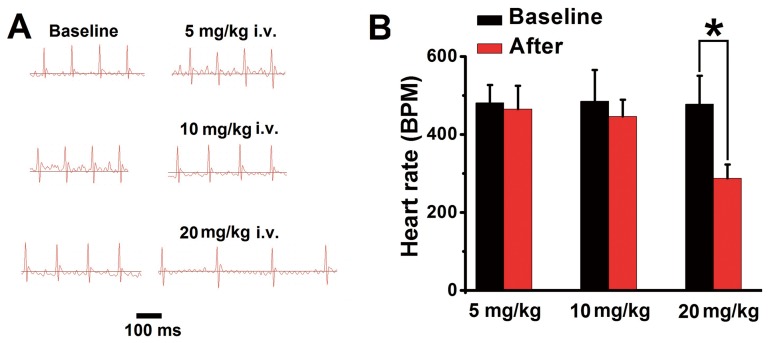
Electrocardiographic (ECG) was recorded before and after intravenous injection of QX-314. A: The ECG trace was captured before (baseline) and three minutes after injection of QX-314. Rats that received 5 mg/kg or 10 mg/kg QX-314 exhibited no significant decrease of heart rate. Rats that received 20 mg/kg QX-314 presented significant decrease of heart rate. B: “After” indicated the heart rate of rats after injection of QX-314. No change of heart rate was found after injection of QX-314 at 5 or 10 mg/kg. QX-314 at dose of 20 mg/kg induced significant decrease of heart rate (n = 4 for analysis and other 6/10 rats died within 3 min after injection). * *P* <0.001, compared with baseline.

As shown in Figure 3 and Table 4, after injection of QX-314 at dose of 5 mg/kg, stable heart rate of rats was 465±60 beats/min, which was similar to its baseline (481±46 beats/min, *P* = 0.546). After injection of QX-314 at dose of 10 mg/kg, stable heart rate of rats was 446±43 beats/min, which was also unchanged compared to baseline (485±80 beats/min, *P* = 0.146). However, significant bradyarrhythmia was found after injection of QX-314 at dose of 20 mg/kg. Three minutes after injection, stable heart rate of rats decreased to 287±35 beats/min in 4/10 rats (*P*<0.001 vs. baseline) and other 6 out of 10 rats died.

**Table 4:** Toxicity of QX-314 on cardiac system in rats.

Dose (mg/kg)	Baseline HR	After HR	*P*-value	Death
5	481±46	465±60	0.546	0/10
10	485±80	446±43	0.146	0/10
20	478±73	287±35*	<0.001	6/10

Results were expressed as mean ± SD. “Baseline HR” was baseline heart rate of rats and “After HR” was heart rate of rats after injection of QX-314. “*P*-value” was the difference between baseline and after injection of QX-314.

*: compared to baseline HR, *P*<0.001.

## Discussion

In the present study, QX-314, a quaternary lidocaine derivative, produced long-lasting and reversible IV regional anesthesia in tail of rats. This regional anesthetic effect of QX-314 was mediated by activation of TRPV1 channels because capsazepine (TRPV1 channel antagonist) significantly reduced the effect of QX-314. By previous studies, QX-314 has been demonstrated to produce nociceptive blockade, sensory blockade and motor blockade in various animal models [11], [12], [13], [14]. Unfortunately, anesthetic potency of QX-314 is attenuated by its positive charge because QX-314 is difficult to penetrate neural membranes [12]. Previous studies also indicated that activation of TRPV1 channels could specifically deliver QX-314 into nociceptor, so as to produce a rapid onset and long-lasting nociceptive-selective blockade [12], [13], [14]. In the present study, QX-314 alone produced long-lasting IVRA without any adjuvant drug. At relatively high concentrations (higher than 10 mM on neurons), QX-314 alone has been demonstrated to activate TRPV1 channel [17]. In this study, QX-314 (0.5% = 15 mM) alone might activate TRPV1 channels to enhance effect of QX-314.

Short analgesic duration after releasing of tourniquet is a main limitation of IVRA [1]. The clinical application of IVRA would be significantly improved if a local anesthetic with ability to induce long-lasting analgesia after tourniquet releasing was developed. In the present study, IVRA duration of 0.5% QX-314 was approximately 8-fold longer than that of 0.5% lidocaine. Of note, the tourniquet was released only 10 min after injection of QX-314 and its analgesic effect was maintained for about 6 hours. This finding provides an attractive local anesthetic for IVRA. At least, QX-314 could be an adjuvant drug to lidocaine for inducing a rapid onset and long-lasting IVRA.

Clinical application of QX-314 might be limited by its toxicities to CNS and cardiac system. QX-314 was considered to be less toxic than lidocaine because quaternary agents are unable to cross blood-brain barrier. By previous study, toxicities of QX-314 to CNS and cardiac system were similar or even higher than that of lidocaine [20]. EC_50_ of QX-314 in rat tail IVRA was 0.15±0.02%, which was similar to the EC_50_ of lidocaine (0.18±0.07%) in rat tail IVRA [22]. For the most common toxic effect of lidocaine, convulsive (tonic-clonic seizure) threshold of lidocaine was 23.5±2.2 mg/kg in rats [25]. Compared to the present study, QX-314 at 20 mg/kg induced tonic-clonic seizure (score 5) in 4/10 rats. Thus, therapeutic window of lidocaine and QX-314 might be similar.

In this study, no obvious toxic complications were observed after tourniquet releasing in all the study rats. For evaluation of toxicities of QX-314 to CNS and cardiac system, toxic effects of QX-314 might be enhanced by direct injection into systemic circulation without any tourniquet. Systemic injection of QX-314 at a dose of 5 mg/kg did not induce any toxic reaction in all the rats. When 10 mg/kg QX-314 (0.5% QX-314 0.5 ml for a rat weighing 250 g) was injected, only light neural symptom was found in 2/10 rats and no cardiac outcome was observed. Therefore, 0.5% QX-314 might be generally safe for IVRA even without tourniquet. Based on these data, low but relevant doses of QX-314 did not result in any measurable toxic effects to CNS and cardiac system. However, injection of 20 mg/kg QX-314 (1% QX-314 0.5 ml for a rat weighing 250 g) was lethal. For EC_50_ of QX-314 in rat tail IVRA, 0.15% QX-314 0.5 ml equals to 3 mg/kg for a rat weighting 250 g, which is far lower than its toxic doses. Therefore, the possibility of QX-314 in IVRA is not ruled out by its toxicity.

According to our previous studies [21], [22], both tail-flick and tail-clamping tests were used, but with different purposes. The tail-clamping test was used to determine rat tail IVRA because it is a standard evaluation for anesthetic and/or nociceptive effect in rats and its result is in an “all-or-none” pattern. At the same time, tail-flick test was used to quantitatively measure the analgesic effect of QX-314 and lidocaine in IVRA, expressed as %MPE. Tail-flick latency increased by 50% was regarded as analgesia while only complete no response to tail-clamping stimulus was regarded as IVRA. Thus, duration of tail-flick blockade was significantly longer than duration of tail-clamping blockade. If only %MPE up to 100% was regarded as effective, the duration of tail-flick blockade would be similar to the duration of tail-clamping blockade. On mechanistic level, tail-clamping test represents the blockade of high thresholds mechanical stimulus, and tail-flick test represents the blockade of thermal stimulus. Both of high thresholds mechanical and thermal stimuli could evoke painful sensation, but they are probably conducted by different types of nervous fibers. By our previous study about IVRA model in rats [21], tail-flick latency would be prolonged if tourniquet maintained for more than 10 min. In clinical setting, duration of tourniquet is far more than 10 min, thus, it is reasonable to believe that duration of IVRA by QX-314 could be even longer than that in rats.

There are some limitations in the present study: firstly, motor blockade in IVRA by QX-314 was not determined because of the tail model. Thus, we could not directly prove the effect of OX-314 was nociceptive-preferred. However, it is rational to expect that the long-lasting IVRA produced by QX-314 might be nociceptive-preferred because IVRA of QX-314 is mediated by activation of TRPV1 channels. TRPV1 channel is exclusively expressed on nociceptor of peripheral nervous system [12]. Further study might be designed to investigate the effects of QX-314 on sensory and motor functions in limb IVRA model of large animals. Secondly, to install ECG leads, 2% isoflurane was applied. Isoflurane alone might affect cardiovascular system. Thus, we could not completely rule out the effect of general anesthesia. Thirdly, tissue and nervous injury induced by local anesthetics is an increasing concern of regional anesthesia. Although all the study rats completely recovered after IVRA, further study about toxicity of QX-314 on tail nerves are needed.

In summary, QX-314 could produce long-lasting and reversible IVRA in a rat model. Anesthetic potency of QX-314 in rat tail IVRA is acceptable compared to its toxic doses on CNS and cardiac system. Our finding indicates the possibility that quaternary local anesthetics may produce clinically useful IVRA.
